# Feasibility of Stress Wave-Based Debond Defect Detection for RCFSTs Considering the Influence of Randomly Distributed Circular Aggregates with Mesoscale Homogenization Methodology

**DOI:** 10.3390/ma16083120

**Published:** 2023-04-15

**Authors:** Jiang Wang, Bin Xu, Qian Liu, Ruiqi Guan, Xiaoguang Ma

**Affiliations:** 1 College of Civil Engineering, Huaqiao University, Xiamen 361021, China; jiangwang@hqu.edu.cn (J.W.); hqulq@stu.hqu.edu.cn (Q.L.); 15632@hqu.edu.cn (R.G.); 2 Key Laboratory for Intelligent Infrastructures and Monitoring of Fujian Province, Huaqiao University, Xiamen 361021, China; 3 Foshan Graduate School, Northeastern University, Foshan 528311, China; maxg@mail.neu.edu.cn; 4 State Key Laboratory of Synthetical Automation for Process Industries, Northeastern University, Shenyang 110819, China

**Keywords:** piezoelectric lead zirconate titanate (PZT), coarse aggregate, concrete mesoscale modeling, rectangular concrete-filled steel tube (RCFST), coupling mesoscale finite element models (CMFEMs), coupling homogenization finite element model (CHFEM), stress wave field, wavelet packet analysis, RAE size effect

## Abstract

In order to efficiently investigate the effect of the mesoscale heterogeneity of a concrete core and the randomness of circular coarse aggregate distribution on the stress wave propagation procedure and the response of PZT sensors in traditional coupling mesoscale finite element models (CMFEMs), firstly, a mesoscale homogenization approach is introduced to establish coupling homogenization finite element models (CHFEMs) with circular coarse aggregates. CHFEMs of rectangular concrete-filled steel tube (RCFST) members include a surface-mounted piezoelectric lead zirconate titanate (PZT) actuator, PZT sensors at different measurement distances, a concrete core with mesoscale homogeneity. Secondly, the computation efficiency and accuracy of the proposed CHFEMs and the size effect of representative area elements (RAEs) on the stress wave field simulation results are investigated. The stress wave field simulation results indicate that the size of an RAE limitedly affects the stress wave fields. Thirdly, the responses of PZT sensors at different measurement distances of the CHFEMs under both sinusoidal and modulated signals are studied and compared with those of the corresponding CMFEMs. Finally, the effect of the mesoscale heterogeneity of a concrete core and the randomness of circular coarse aggregate distribution on the responses of PZT sensors in the time domain of the CHFEMs with and without debond defects is further investigated. The results show that the mesoscale heterogeneity of a concrete core and randomness of circular coarse aggregate distribution only have a certain influence on the response of PZT sensors that are close to the PZT actuator. Instead, the interface debond defects dominantly affect the response of each PZT sensor regardless of the measurement distance. This finding supports the feasibility of stress wave-based debond detection for RCFSTs where the concrete core is a heterogeneous material.

## 1. Introduction

### 1.1. Need and Background

As typical structural components carrying increasing vertical or axial load, large-scale concrete-filled steel tube (CFST) members have been extensively applied as columns in high-rise buildings or arches and piers in long-span bridges. The non-uniformly distributed temperature in the curing procedure of the concrete core of CFST members after pouring concrete and the unavoidable long-term shrinking and creeping of concrete core may cause interface debond defects between the concrete core and steel tube, weakening the desired confinement effect of the steel tube on the concrete core and negatively affecting the ductility and load-carrying capacity of the CFST members. Therefore, the development of reliable interface debond defect detection methodologies for the CFST members is of great emergency. Most of the traditional non-destructive testing (NDT) techniques, which have been successfully applied in defect detection for reinforced concrete (RC) or steel structures, including infrared thermal imaging [1,2], electromagnetic method [3,4], and ultrasonic and acoustic emission [5,6,7], have difficulties in detecting interface debond defects for CFSTs due to the electromagnetic shielding effects of the steel tube, its structural complexity, and the huge difference in material parameters of concrete and steel.

### 1.2. State of the Art 

Recently, defect detection for concrete materials, composites, RC and steel structures based on stress wave measurement using piezoelectric lead zirconate titanate (PZT) actuators and sensors have attracted much attention [8,9,10,11,12,13]. Gu et al. [14] and Yan et al. [15] monitored cracks in RC columns using multi-functional smart aggregates (SAs) and PZT patches. Wu and Chang performed an experimental study for the purpose of monitoring debond between steel reinforcement and concrete in RC structures using embedded PZT sensors and actuators [16]. By using PZT sensors, an electromechanical impedance (EMI) method for detecting the shear connection bolt loosening of a steel-concrete composite girder was developed and validated experimentally [17]. Markovic et al. [18] numerically studied the traveling procedure of stress waves to understand the mechanism of stress wave-based defect detection for concrete beams with PZT materials. In recent years, embedded SAs and PZT patches have been employed to monitor the condition and to detect defects of CFST members with different cross-section shapes. Using embedded SAs, Zhang et al. monitored a concrete failure procedure near the bottom of L-shaped CFST columns under cyclic loadings [19]. Feng et al. developed an active sensing method using embedded SAs to monitor the grouting quality of a CFST arch bridge and demonstrated the feasibility of the proposed method [20].

One of the most potential defects in CFST members is the interface debond defect between the concrete core and steel tube. In order to accurately detect interface debond defects in CFST members, Xu et al. [21,22] developed an active approach by employing PZT patches as either actuators or sensors and using the wavelet packet energy of different embedded PZT sensor measurements for the first time. For the convenience of the PZT actuator and sensor installation, Chen et al. [23] proposed an interface debond defect detection approach with multi-channel analysis of surface waves (MASW) for CFSTs and illustrated the feasibility of the proposed method both experimentally and numerically. In order to numerically investigate the mechanism of stress wave measurement-based debond detection for CFST members efficiently, a spectral element method (SEM) was developed by Xu et al. and Luan et al. to simulate local stress wave propagation and the response of an embedded PZT sensor in a CFST substructure [24,25]. The relationship between the debond dimension in a circular CFST column under both sinusoidal and swept frequency excitation signals and the corresponding amplitude and wavelet packet analysis of output signals collected by a PZT sensor was studied in detail [26,27,28]. Considering the direct and the inverse piezoelectric effects of PZT materials and the coupling effect between the PZT materials and the CFST members, Chen et al. [29] constructed a multi-physics coupled finite element method (FEM) composed of surface-mounted PZT actuators, embedded PZT sensors and a rectangular CFST column, and elucidated the relationship between input voltage amplitudes and PZT sensor measurements and the influences of different frequencies and measurement distances on each PZT response and on the stress wave fields of CFSTs with and without interface debond defects.

However, one of the main concerning shortages in the above-mentioned numerical analysis on stress wave travel in CFST members is that the concrete core was considered as a homogeneous material with uniform material parameters. Considering the fact that concrete is a typical heterogeneous material, composed of mortar, coarse and fine aggregates and an interfacial transition zone (ITZ) between aggregates and the mortar, etc., it has been a common concern if the mesoscale heterogeneity and randomness of aggregate distribution in the concrete core dominantly affect stress wave travel within CFSTs and PZT sensor responses at different measurement distances when compared with interface debond defects in CFSTs [30]. With the help of the random aggregate method (RAM) for modeling mesoscale concrete materials, Xu et al. [31] numerically investigated the influence of the mesoscale concrete core in RCFSTs on stress wave fields and the response of a PZT sensor with a certain measurement distance and compared the influence of both an interface debond defect and the mesoscale heterogeneity and randomness of the concrete core on stress wave measurement. Unfortunately, numerical simulation of stress wave propagation in CFSTs using mesoscale models is extremely time-consuming because very fine finite elements are usually required. Therefore, the development of mesoscale concrete modeling and stress wave propagation simulation approaches for CFST members with high computation efficiency is highly desired.

The mesoscale homogenization method is computationally efficient for the behavior simulation of mesoscale concrete materials and structures, where the mesoscale concrete is modeled with a number of representative area elements (RAEs) two-dimensionally (in 2D) or representative volume elements (RVEs) three-dimensionally (in 3D). The concrete material of each RVE or RAE is homogenized with uniform mechanical properties [32,33,34]. Wang et al. [35] preliminarily explored the effect of the mesoscale concrete core on stress wave travel and the response of an embedded PZT sensor at a certain measurement distance of CFSTs modeled with RAEs for the first time, where the debond defect was found to be the dominant factor affecting the stress wave propagation instead of the mesoscale heterogeneity and randomness of concrete aggregate distribution.

### 1.3. Research Significance

In the study by Wang et al. [35], the effects of the dimension of RAEs on computational efficiency and accuracy and the shape of the randomly distributed circular aggregates on the stress wave field and the responses of PZT sensors at different measurement distances in RCFSTs have not been thoroughly investigated. Further investigation into the effects of different RAE dimensions of mesoscale homogenization models and of the random distribution of aggregates on the stress wave fields and PZT sensor measurements at different measurement distances is highly needed. Additionally, after confirming the influence of the mesoscale heterogeneity and randomness of concrete on stress wave propagation and different PZT sensor responses, a comparison between it and that of interface debond defects can be made. The finding from this study illustrates the feasibility of the interface debond detection method using stress wave measurement for CFSTs where concrete is inherently heterogeneous at the mesoscale. Moreover, studying the influence of the mesoscale heterogeneity and randomness of a concrete core on PZT sensor responses at different measurement distances is also helpful for practical PZT sensor arrangement for the debond detection of CFSTs in practice.

### 1.4. Research in This Paper

In this paper, multi-physics coupling mesoscale finite element models (CMFEMs) composed of a surface-mounted PZT actuator, a surface-mounted or embedded PZT sensor, and a 2D mesoscale RCFST specimen with randomly distributed circular aggregates are firstly established. After that, the corresponding coupling homogenization finite element models (CHFEMs) with different RAE dimensions are established to study the effect of RAE dimensions on both stress wave fields and the response of PZT sensors at different measurement distances. For simplicity, the stress wave fields in mesoscale finite element models (MFEMs) and the corresponding homogenization finite element models (HFEMs) with and without debond defects are determined considering different RAE dimensions. The effects of RAE dimensions on the simulation results of stress wave fields are analyzed comprehensively. Therefore, the PZT sensor responses with different measurement distances in CMFEMs and the corresponding CHFEMs with and without debond defects under various excitation signals are also determined considering different RAE dimensions. The results show that the mesoscale heterogeneity of a concrete core and the randomness of circular aggregate distribution only have a certain influence on the response of PZT sensors that are close to the surface-mounted PZT actuator. Compared with the mesoscale heterogeneity of a concrete core in the form of the random distribution of circular aggregates, the interface debond defect dominantly affects the stress wave fields and the response of PZT sensors at various measurement distances.

## 2. Multi-Physics CMFEMs for RCFST–PZT Coupling Model with the RAM Approach

In recent years, the effect of the morphology of coarse aggregates on the properties of concrete materials and structures has been an active topic [36]. In this study, considering the mesoscale heterogeneity of concrete cores in RCFSTs, CMFEMs for RCFST–PZT coupling models consisting of mortar, randomly distributed coarse and fine circular aggregates, steel tube and PZT patches as either actuators or sensors are constructed. The stress wave fields and the responses of PZT sensors at various measurement distances for RCFSTs considering the mesoscale heterogeneity of concrete cores and randomness of circular coarse aggregate distribution are explored numerically.

The piezoelectric equation for the employed PZT patches can be found in the previous studies [23,24,37,38,39]. The general finite element equation dealing with the dynamic response of CFST members can be expressed as in Equation (1) [37].

(1) $$\left[M\right]\left\{\ddot{\mu }\right\}+\left[C\right]\left\{\dot{\mu }\right\}+\left[K\right]\left\{\mu \right\}=\left\{F\right\}$$

where $\left[M\right]$ represents the mass matrix, $\left[K\right]$ is the matrix of stiffness, $\left[C\right]$ denotes the damping matrix, $\left\{\mu \right\}$ stands for the displacement vector of the CFST member, and $\left\{F\right\}$ represents the load vector applied to it.

In addition, the equation of motion for the coupled RCFST–PZT model is described in the form of vectors and generalized matrices [27,35]:

(2) $$\left[\begin{array}{cc} \left[M\right] & \left[0\right]\\ \left[0\right] & \left[0\right] \end{array}\right]\left\{\begin{array}{c} \left\{\ddot{\mu }\right\}\\ \left\{\ddot{V}\right\} \end{array}\right\}+\left[\begin{array}{cc} \left[C\right] & \left[0\right]\\ \left[0\right] & \left[0\right] \end{array}\right]\left\{\begin{array}{c} \left\{\dot{\mu }\right\}\\ \left\{\dot{V}\right\} \end{array}\right\}+\left[\begin{array}{cc} \left[K\right] & \left[K^{Z}\right]\\ {\left[K^{Z}\right]}^{T} & \left[K^{d}\right] \end{array}\right]\left\{\begin{array}{c} \left\{\mu \right\}\\ \left\{V\right\} \end{array}\right\}=\left\{\begin{array}{c} \left\{F\right\}\\ \left\{Q\right\} \end{array}\right\}$$

where $\left[K^{d}\right]$ represents the dielectric matrix, $\left[K^{Z}\right]$ is the electromechanical coupling matrix, and {Q} and $\left\{V\right\}$ are the quantity of electric charge and the electric potential on the electrode surface, respectively.

In this study, the plane dimensions of the concrete core of the 2D RCFST member are 400 mm by 400 mm and the thickness of the concrete core is 10 mm. The concrete core is surrounded by a plane steel tube with a thickness of 5 mm. The employed PZT patches acting as either sensors or actuators are of a length of 10 mm and a thickness of 0.3 mm. The polarization direction of each PZT patch is its thickness direction. In order to investigate the influence of different measurement distances between the PZT actuator and the PZT sensors, a number of mesoscale RCFST–PZT coupling models with different PZT sensor locations are established with the RAM approach [31,35].

The size and quantity of circular aggregates in a specific RCFST member are specified according to an ideal gradient curve shown as follows [31].

(3) $$P(D<D_{0})=\sqrt{D/D_{max}}$$

where $P(D<D_{0})$ is the percentage of the volume occupied by aggregates with the diameter being D < $D_{0}$, and $D_{0}$ and $D_{max}$ are the diameter of the sieve pore and the maximum particle size of aggregates, respectively.

Since wave propagation simulation in RCFST members is time-consuming in 3D numerical simulation, the fuller gradation curve is converted into 2D according to the following Equation (4) with the Walraven method.

(4) $$P(D<D_{0})=P_{k}\left[1.065{\left(\frac{D_{0}}{D_{max}}\right)}^{0.5}-0.053{\left(\frac{D_{0}}{D_{max}}\right)}^{4}-0.012{\left(\frac{D_{0}}{D_{max}}\right)}^{6}-0.0045{\left(\frac{D_{0}}{D_{max}}\right)}^{8}+0.0025{\left(\frac{D_{0}}{D_{max}}\right)}^{10}\right]$$

where $P_{k}$ is the percentage of the volume occupied by aggregates of a diameter of D < $D_{0}$.

In a Cartesian coordinate system, the coordinates of the center of a circle are randomly generated within the RCFST member using the Monte Carlo method. In order to avoid circular aggregate overlap and contact, the distance between any two circular aggregates must be greater than a certain value. The overlap of two circular aggregates is judged based on the distance between their centers according to Equation (5). The aggregate with a large radius should be put in first because the effective delivery area after the successful delivery of an aggregate is gradually reduced.

(5) $$\sqrt{{\left(x_{j}-x_{i}\right)}^{2}+{\left(y_{j}-y_{i}\right)}^{2}}>\alpha \left(r_{j}+r_{i}\right)$$

where ($x_{i}$, $y_{i}$) and ($x_{j}$, $y_{j}$) are the coordinates of the centers of the generated i-th and j-th circular aggregates, respectively, $r_{i}$ and $r_{j}$ denote the radius of the *i*-th and *j*-th circular aggregates and α takes the value of 1.05 in this paper.

**Figure 1:**
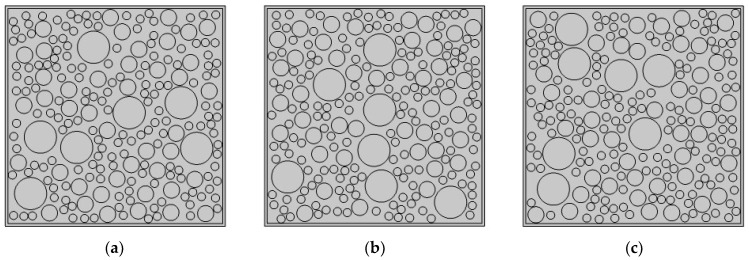
Mesoscale concrete core in RCFSTs using RAM. (**a**) CMFEM 1; (**b**) CMFEM 2; (**c**) CMFEM 3.

**Figure 2:**
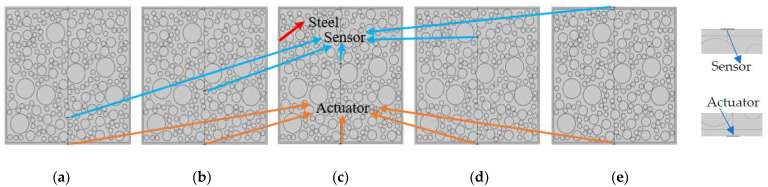
CMFEM 1 with PZT sensors at different measurement distances: (**a**) 80 mm; (**b**) 160 mm; (**c**) 240 mm; (**d**) 320 mm; (**e**) 410 mm.

Here, three CMFEMs with different randomly distributed circular coarse aggregates mimicking the mesoscale heterogeneity and randomness of a concrete core, i.e., CMFEM 1, 2 and 3, are established as shown in Figure 1. In each CMFEM model, five PZT measurement distances, 80 mm, 160 mm, 240 mm, 320 mm and 410 mm, are considered. Figure 2 shows the mesoscale models of CMFEM 1 with the same randomly distributed circular coarse aggregates but different PZT sensor measurement distances. The material property of mortar, steel tube, circular coarse aggregates and the PZT patches of the mesoscale model are listed in Table 1 [32].

## 3. Multi-Physics CHFEMs for CMFEMs of RCFST–PZT Coupling Structures with Circular Coarse Aggregates

As a further investigation of the previous study by Wang et al. [35], CHFEMs with different RAE dimensions corresponding to each CMFEM are established with the help of a mesoscale equivalent homogenization approach [32,33,35]. By comparing the stress wave field simulation results using the CHFEMs with different RAE dimensions and those of the corresponding CMFEM 1, the influence of RAE dimensions on the stress wave propagation simulation results within the RCFST members with and without a debond defect is investigated, where the debond between concrete and steel tube is mimicked by air. In addition, the response of each PZT sensor at the various measurement distances of CMFEMs is also compared with that of the corresponding CHFEMs with different RAE dimensions. All numerical simulations are conducted in COMSOL as a solver.

### 3.1. Meshing for Both CMFEMs and the Corresponding CHFEMs

In order to study the computational accuracy of the CHFEMs, CHFEMs with different RAE dimensions are established for each CMFEM. The material properties including density, Poisson’s ratio and elastic modulus of each RAE with different dimensions can be determined according to the method proposed by Wang et al. [35], where each homogenized RAE consists only of mortar and circular coarse aggregates since the ITZ has a minor impact on the measurement results made by the PZT sensor. By the use of the mesoscale equivalent homogenization approach, the values of the material parameters of each RAE are determined but not listed here due to space limitations. It is worth noting that the material parameters for each RAE are different from each other in each CHFEM because of the different coarse aggregate and mortar contents in each RAE. The material of each RAE is assumed to be elastic and homogeneous for stress wave propagation simulation. Moreover, the surfaces between different materials including those between PZT patches and steel tubes and those between concrete cores and steel tubes share common nodes to maintain displacement and deformation compatibility. In the mesoscale simulation of stress wave fields and the responses of different PZT sensors, the selection of the maximum element dimension and the integration time step is critical, and is determined according to the input signal frequency. The maximum element dimension and integration time step should meet the requirements described in reference [26].

**Figure 3:**
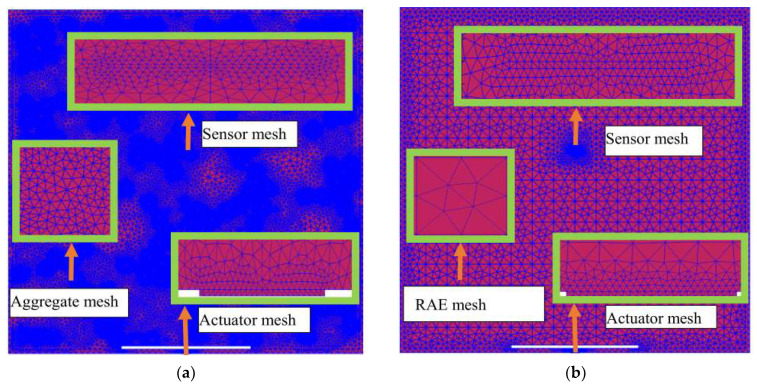
Meshing of the mesoscale RCFST–PZT coupling model and its corresponding homogenization model when the frequency of excitation is 40 kHz. (**a**) CMFEM 1; (**b**) CHFEM with RAE dimension of 25 mm by 25 mm.

For the debond defect detection of RCFST members using stress wave fields and PZT sensor response measurements, high-frequency signals are commonly used as inputs on a PZT actuator mounted on the surface of the steel tube of an RCFST. The high-frequency excitation signal leads to a short stress wave wavelength, where fine meshing and short integration time steps are required. Figure 3a shows an example of the meshing of the CMFEM 1 where the excitation signal frequency is 40 kHz, and Figure 3b shows the meshing of the corresponding CHFEM, where the size of the RAE is 25 mm × 25 mm. It can be seen from Figure 3a that CMFEM 1 has more elements than the corresponding CHFEM.

Here, the typical 2D RAE dimensions of 20 mm by 20 mm, 25 mm by 25 mm and 40 mm by 40 mm for each CMFEM model are considered to distinguish the size effect of RAEs on the stress wave fields and PZT sensors measurement simulation results. Since the advantage of the computational efficiency of CHFEM has been demonstrated in the recent research by Wang et al. [35], a comparison of the computational efficiency between each CHFEM and the corresponding CMFEMs is not shown here. The electrical boundary of PZT patches and the mechanical boundary of each CHFEM and CMFEM are defined in accordance with the previous study by Chen et al. [23] and Xu et al. [28].

### 3.2. Excitation Signals

For the mesoscale simulation of the stress wave field within different mesoscale RCFSTs, it is convenient to exclude the direct and inverse piezoelectric effects of PZT actuators and sensors and the coupling effect between them and the RCFST member. In this study, the stress wave propagation procedure within different mesoscale finite element models (MFEMs) of RCFSTs and the corresponding homogenization finite element models (HFEMs) employing different RAE sizes under the excitation of a pulse force are investigated for comparison.

The applied pulse force signal for stress wave fields and modulated sinusoidal excitation signal for the PZT sensor measurement simulation is the same as that in [31]. The modulated sinusoidal excitation signal for the PZT sensor measurement simulation is listed below.

(6) $$V_{t}=V_{0}\sin\left(2\pi ft\right)\sin{\left(\frac{2\pi ft}{10}\right)}^{2}$$

where $V_{t}$ is the input voltage signal and $V_{0}$ represents the amplitude of the input signal, the value of which is 10 V. f stands for the 20 kHz frequency of the input signal. t is the time instant and takes the value of 2–7 s.

The measurement results of different PZT sensors under both sinusoidal and modulated signals are simulated using both the CMFEMs and the corresponding CHFEMs, where the circular coarse aggregates are randomly distributed and the concrete core is heterogeneous at the mesoscale.

## 4. RAE Dimension Effect on Stress Wave Propagation Simulation Results

In the study by Wang et al. [35], the influence of different RAE dimensions on the simulation results of the stress wave fields in homogenization finite element models (HFEMs) is not considered. In this section, the stress wave fields in the form of the amplitude of the displacement of mesoscale finite element models (MFEMs) with and without debond defects and their corresponding HFEMs with different RAE dimensions are discussed. Without loss of generality, the MFEM 1 with identical mesoscale structure to the CMFEM 1 as shown in Figure 1a and the corresponding HFEMs with different RAE dimensions are studied.

### 4.1. Stress Wave Fields for the Healthy MFEM 1 and Its Corresponding HFEMs with Different RAE Dimensions

Without a loss of generality, the stress wave fields of the healthy MFEM 1 without interface debond defects and the corresponding HFEMs with different RAE dimensions at different time instants are investigated and compared at first. The three side dimensions of 20 mm, 25 mm and 40 mm for square RAEs are considered.

**Figure 4:**
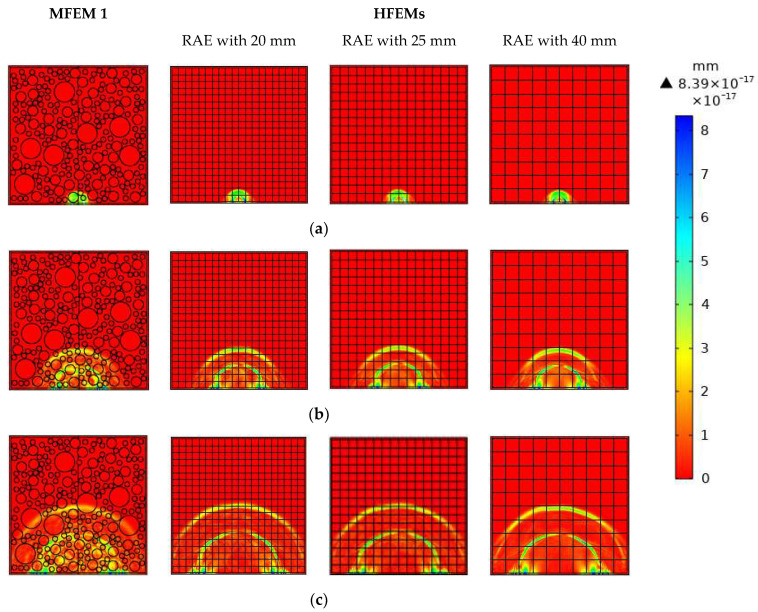
Stress wave fields of MFEM 1 and its corresponding HFEMs with different RAE dimen-sions at different times: (**a**) t = 1 × 10^−5^ s; (**b**) 3 × 10^−5^ s; (**c**) t = 5 × 10^−5^ s.

In Figure 4, a comparison of the stress wave fields of the healthy MFEM 1 and the corresponding three HFEMs with different RAE dimensions at different time instants is illustrated. The pulsed force signal at the frequency of 100 kHz is used to excite the RCFST member at the location of the PZT actuator. From Figure 4, the stress wave fields of the healthy MFEM 1 and its corresponding HFEMs with different RAE dimensions maintain high consistency at each identical time instant. It is clear that the three HFEMs with three different RAE dimensions can simulate the stress wave fields accurately. The stress wave fields of each HFEM are close to each other and also are very close to those of MFEM 1. Moreover, through homogenization at the mesoscale, the stress wave fields of the HFEMs are much clearer than those of the corresponding MFEM.

In addition to a comparison of the element numbers, DOFs and the computation time for stress wave propagation simulation between the healthy MFEM 1 and its corresponding HFEMs with different RAE dimensions are shown in Table 2. The computer workstation used for the simulation has two high-performance Intel Gold 6130 central processing units (CPUs) with a total of 32 cores and 128 GB of random-access memory. It is clear that the simulation time of HFEMs is about 3000 s less than that of the MFEM. By replacing the traditional MFEM with the HFEM, the improvement in computational efficiency is very obvious, although the difference in the computational efficiency of stress wave propagation within HFEMs with different RAE dimensions is not significant. With the increase in the RAE dimension, the number of FEMs and the corresponding simulation time decrease.

### 4.2. Stress Wave Fields for the MFEM 1 with an Interface Debond Defect and Its Corresponding HFEMs with Different RAE Dimensions

**Figure 5:**
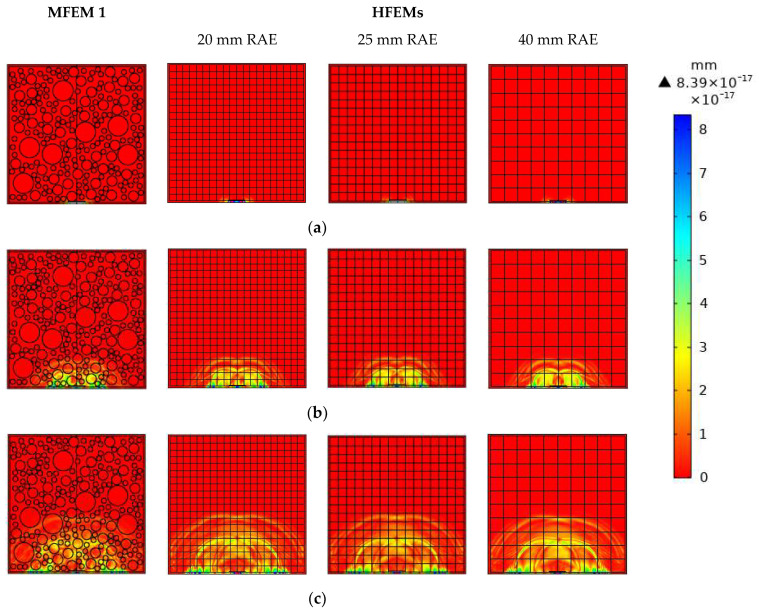
The stress wave fields of both the multi-scale CFST model with a debond defect and its equivalent HFEMs with different RAE dimensions at different time instants: (**a**) t = 1 × 10^−5^ s; (**b**) 3 × 10^−5^ s; (**c**) t = 5 × 10^−5^ s.

In order to further investigate the effect of different RAE dimensions on the stress wave field simulation results and to compare it with that of the interface debond defect, the stress wave fields of the MFEM 1 with a debond defect and the corresponding HFEMs with different RAE dimensions at different time instants are simulated. As shown in Figure 5, the interface debond defect in the MFEM 1 and HFEMs is located at the center of the bottom and is of a length of 50 mm along the steel tube and a depth of 4 mm. Here, the side dimensions of the square RAEs of the HFEMs corresponding to MFEM 1 take the values of 20 mm, 25 mm and 40 mm, respectively.

The stress wave fields of the MFEM 1 with an interface debond defect and its corresponding HFEMs with different RAE dimensions are shown and compared in Figure 5. Similarly to the findings in Figure 4, it is clear that at each identical time instant, the stress wave fields of the MFEM 1 with a debond defect and its corresponding HFEMs with different RAE dimensions maintain high consistency. Comparing the stress wave fields of different HFEMs using different RAE dimensions, the RAE dimension has no obvious effect on the stress wave field simulation results. Similarly, the stress wave fields of the HFEMs are clearer than those of the corresponding MFEM 1 due to its homogenization.

When comparing the stress wave fields of the healthy RCFSTs at each identical time instant shown in Figure 4 to those of the RCFST with an interface debond defect shown in Figure 5, it can be found that the debond defects in the RCFSTs significantly affect the propagation of stress waves in MFEM 1 and HFEMs. The interface debond defect dominantly delays the stress wave travel and attenuates the amplitude of the wave propagation.

In practice, for the debond detection of RCFST members, it is difficult to get the stress wave fields at different time instants. PZT sensors are usually used to measure the stress wave at certain locations and the measurements are used to detect the debond defect. Therefore, a further numerical study on the response of PZT sensors in CMFEMs and the CHFEMs at different measurement distances is carried out to distinguish the influences of the mesoscale heterogeneity of concrete on PZT sensor measurement and those of debond defects in the following chapter.

## 5. PZT Sensor Measurements of CMFEMs with Randomly Distributed Circular Coarse Aggregates and the Corresponding CHFEMs

The effects of both themesoscale heterogeneity of concrete cores in the form of randomly distributed circular coarse aggregates and debond defects on the output of PZT sensors at different measurement distances from the PZT actuator are investigated. Multi-physics field simulation on the measurement of PZT sensors of three CMFEMs and the corresponding CHFEMs with different RAE dimensions under both sinusoidal excitation and modulated excitation is performed. The frequency of the sinusoidal input signal is 20 kHz and the frequency of the modulated excitation signal described in Equation (3) is 20 kHz. Both input signals are of an amplitude of 10 V. The distances between the PZT sensor and actuator are assigned to be 80 mm, 160 mm, 240 mm, 320 mm and 410 mm. The distance of 410 mm means that the PZT sensor is installed on the opposite side surface of the RCFST, representing the surface-mounted PZT actuator and sensor installation strategy. Considering the similarity of the stress wave fields of the HFEMs with different RAE dimensions shown in Figure 4 and Figure 5, here, the PZT sensor response results for the three CMFEMs and the corresponding CHFEMs with a RAE dimension of 25 mm are not presented due to space limitations.

### 5.1. Comparison of PZT Sensor Responses at Different Measurement Distances of CMFEMs

#### 5.1.1. PZT Sensor Measurements under Sinusoidal Input Signal of CMFEMs

**Figure 6:**
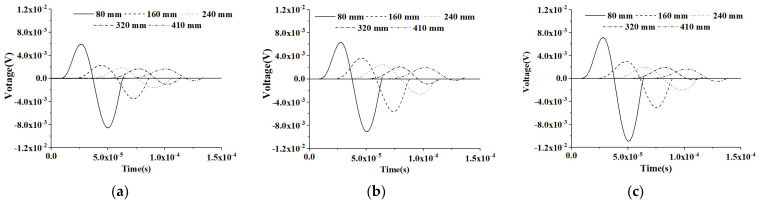
Time-domain response of sensors at different measurement distances from the actuator under a sinusoidal excitation signal for healthy CMFEMs. (**a**) CMFEM 1; (**b**) CMFEM 2; (**c**) CMFEM 3.

Figure 6 shows the time-domain response of the PZT sensors at different measurement distances of the three healthy CMFEMs under the sinusoidal input signal. The results show that the mesoscale heterogeneity of concrete cores and the randomness of coarse aggregate distribution in CMFEMs have no significant influence on the response of the PZT sensor at an identical measurement distance. When comparing the time-domain responses of PZT sensors of an identical CMFEM, the time delay of each PZT sensor caused by the increase in measurement distance variations can be observed clearly. Moreover, as the measurement distance of the PZT sensor increases, the attenuation of the voltage amplitude response can be also observed.

**Figure 7:**
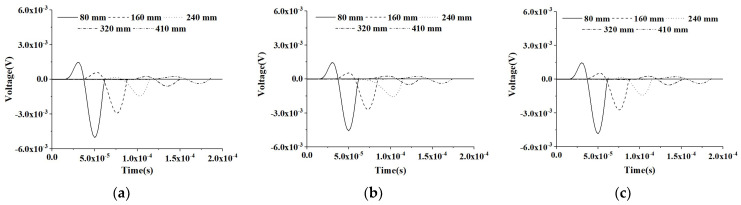
Time-domain response of sensors at different measurement distances from the actuator under a modulated sinusoidal excitation signal for CMFEMs with an interface debond defect. (**a**) CMFEM 1D; (**b**) CMFEM 2D; (**c**) CMFEM 3D.

In order to investigate the effect of interface debond defect on PZT sensor measurements, a numerical simulation on the PZT response of the three CMFEMs with identical debond defects, CMFEM 1D, CMFEM 2D and CMFEM 3D, is also carried out. Figure 7 shows the time-domain signals of the PZT sensors of the three CMFEMs with identical debonds under a sinusoidal input signal. Similarly to the findings in Figure 6, the mesoscale heterogeneity of concrete cores and the randomness of coarse aggregate distribution in the CMFEMs with an interface debond do not affect the output signals of the PZT sensor at an identical measurement distance significantly. From Figure 7, the time delay of each PZT sensor response caused by the increase in the measurement distance can also be clearly observed. Moreover, an attenuation of the amplitude of the PZT sensor can be found as the measurement distance increases.

Comparing the PZT sensor response at identical measurement distance of CMFEMs with and without a debond defect as shown in Figure 6 and Figure 7, the amplitude of PZT sensor response at an identical measurement distance in CMFEM 1D, 2D and 3D with a debond is obviously lower than that of the corresponding healthy CMFEM 1, 2 and 3. Moreover, the stress wave traveling time from the actuator to a PZT sensor of the CMFEMs with debond defects is longer than that of the CMFEMs without debond when the measurement distance is identical. This finding meets the stress wave field results shown in Figure 4 and Figure 5. The interface debond defect has a significant influence on the measurements of PZT sensors and on the randomness of coarse aggregate distribution, and the heterogeneity of a concrete core in a RCFST does not affect the PZT sensor response obviously.

#### 5.1.2. PZT Sensor Measurements under Modulated Sinusoidal Input Signal of CMFEMs

**Figure 8:**
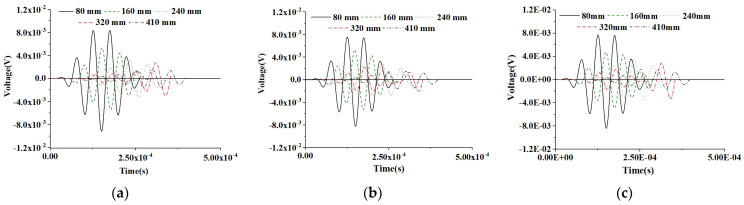
Output voltage collected by sensors at different measurement distances from the actuator under a modulated excitation signal for healthy CMFEMs. (**a**) CMFEM 1; (**b**) CMFEM 2; (**c**) CMFEM 3.

**Figure 9:**
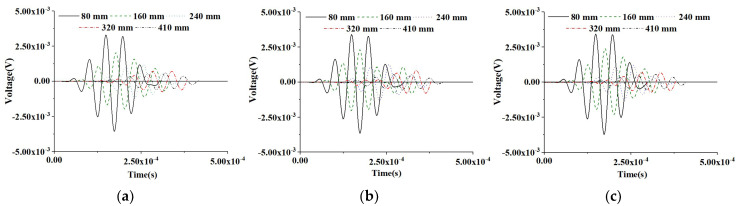
Output voltage collected by sensors at different measurement distances from the actuator under a modulated sinusoidal excitation signal for CMFEMs with an interface debond defect. (**a**) CMFEM 1D; (**b**) CMFEM 2D; (**c**) CMFEM 3D.

In this section, the time-domain responses of PZT sensors at different measurement distances from the PZT actuator of the three CMFEMs with and without an interface debond defect under the modulated sinusoidal excitation signal are investigated and the results are shown in Figure 8 and Figure 9.

The similarity of the PZT sensor measurements at an identical CMFEM measurement distance to different mesoscale structures can be found in Figure 8. Figure 9 shows the comparison of the response of the PZT sensors of the three CMFEMs with an interface debond defect and the different mesoscale structures of the concrete core. It can be found that the heterogeneity of the concrete core and the randomness of coarse aggregate distribution have no obvious influence on the response of each PZT sensor. Moreover, the stress wave traveling time delay and the reduction in the amplitude of the PZT sensor measurement due to the interface debond defect can also be detected clearly.

### 5.2. Comparison of Responses of PZT Sensors with Different CHFEM Measurement Distances with Different RAE Dimensions

In the previous study by the authors of [35], the influence of the mesoscale concrete core of the RCFST on a PZT sensor at a certain distance from the actuator is studied with a CHFEM with a certain RAE dimension. Here, the responses of PZT sensors at the different measurement distances of three CHFEMs with different square RAE side dimensions including 20 mm, 25 mm and 40 mm are further investigated. Without a loss of generality, in this section, the results of the three CHFEMs corresponding to CMFEM 1 are described.

#### 5.2.1. PZT Sensor Measurements under Sinusoidal Input Signal of CMFEMs

**Figure 10:**
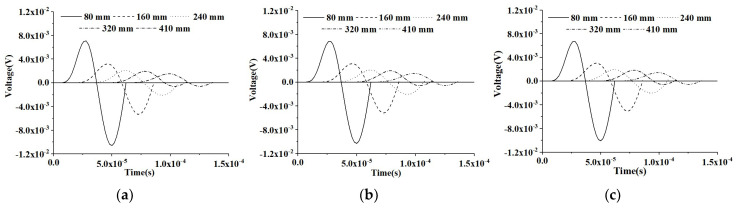
Responses of PZT sensors at different measurement distances from the actuator under sinusoidal excitation of three CHFEMs corresponding to the healthy CMFEM 1 with different RAE dimensions: (**a**) 20 mm; (**b**) 25 mm; (**c**) 40 mm.

Figure 10 shows the time-domain response of PZT sensors at different measurement distances of the healthy CHFEMs corresponding to the CMFEM 1 without an interface debond defect considering different RAE dimensions. It can be found that an increase in the measurement distance of the PZT sensors in the healthy RCFST member leads to a stress wave traveling time delay and amplitude attenuation. However, the RAE dimensions of the three CHFEMs have no significant effect on the amplitude and time instant of the response of the PZT sensor at an identical measurement distance, according to the comparison of the results shown in Figure 10a–c.

**Figure 11:**
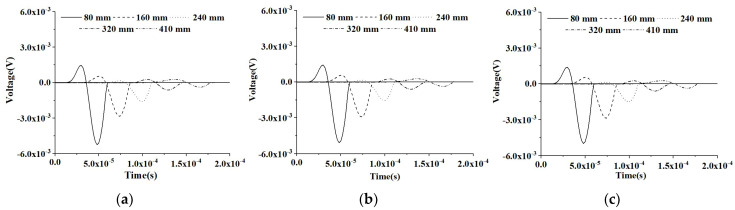
Response of PZT sensors at different measurement distances from the actuator under modulated sinusoidal excitation of CHFEMs with an interface debond corresponding to CMFEM 1 using different RAE dimensions: (**a**) 20 mm; (**b**) 25 mm; (**c**) 40 mm.

To further compare the influence of the debond on the measurement of PZT sensors at different measurement distances, the responses of PZT sensors of the CHFEMs with an interface debond defect using different RAE dimensions are shown in Figure 11. Comparing the response of PZT sensors at identical measurement distances, the difference in the RAE dimension does not have an obvious influence on the measurement results of the PZT sensor with an identical measurement distance. The interface debond defect is still the dominant factor affecting the response of each PZT sensor.

#### 5.2.2. PZT Sensor Measurement under a Modulated CHFEM Sinusoidal Input Signal

**Figure 12:**
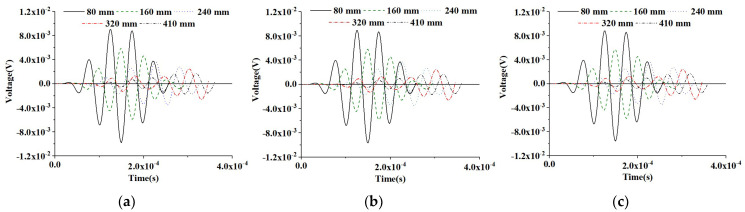
Response of sensors at different measurement distances from the actuator under a modulated sinusoidal excitation signal for healthy CHFEMs using different RAE dimensions: (**a**) 20 mm; (**b**) 25 mm; (**c**) 40 mm.

**Figure 13:**
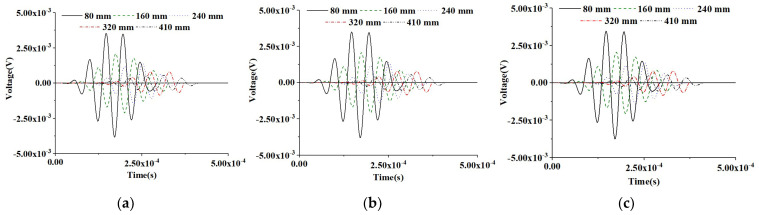
Response of sensors at different measurement distances from the actuator under a modulated excitation signal for CHFEMs with an interface debond defect using different RAE dimensions: (**a**) 20 mm; (**b**) 25 mm; (**c**) 40 mm.

A further investigation on the influence of RAE dimensions of CHFEMs on PZT sensor responses under the modulated sinusoidal excitation signal is performed. Figure 12 displays the time-domain response of PZT sensors at different measurement distances of the healthy CHFEMs with different RAE dimensions. The consistency of PZT sensor responses to different CHFEMs at identical measurement distances and the attenuation and time delay of different PZT sensors at identical CHFEM distances are similar to those described in Section 5.2.1. Figure 13 shows the time-domain responses of PZT sensors for the CHFEMs with an interface debond defect using different RAE dimensions. Comparing the PZT sensor responses shown in Figure 13 with those in Figure 12, it is found that the interface debond defect causes a significant decrease in the response of each PZT sensor at an identical measurement distance.

From all the above-mentioned simulation results of both CMFEMs and CHFEMs, it is concluded that debond defects are the dominant factor affecting the PZT sensor’s response at identical measurement distances. A close look at the influence of the heterogeneity of concrete cores and randomness of coarse aggregate distribution in RCFSTs on the responses of PZT sensors at different measurement distances is taken in a quantitative manner in the following chapter.

## 6. Quantitative Comparison

Further analysis of the maximum amplitude of the time-domain response of the PZT sensors under a sinusoidal input signal and of the wavelet packet energy of the response of PZT sensors under a modulated input signal is carried out.

### 6.1. Responses of PZT Sensors at Different Measurement Distances of Both CMFEMs and CHFEMs

**Figure 14:**
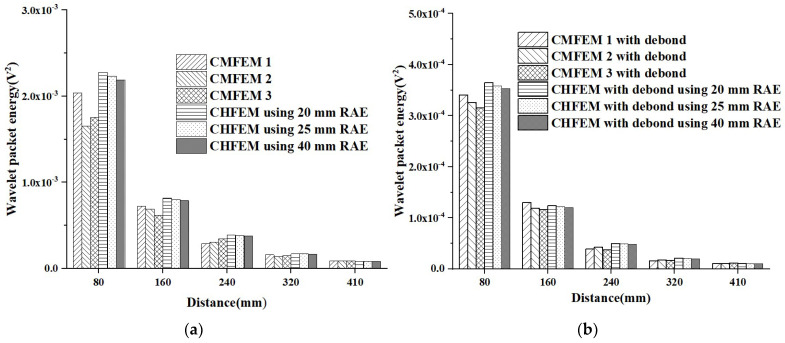
Comparison of the responses of PZT sensors between CMFEMs and CHFEMs corresponding to CMFEM 1 with different RAE sizes under sinusoidal signals. (**a**) Healthy CMFEMs and CHFEMs, and (**b**) CMFEMs and CHFEMs with an interface debond defect.

Figure 14a compares the amplitude of the response of PZT sensors at different measurement distances of the three healthy CMFEMs and of the three CHFEMs corresponding to the healthy CMFEM 1 using different RAE dimensions under the sinusoidal signals. Figure 14b shows the comparison of the amplitude of the response of PZT sensors at different measurement distances of the three CMFEMs and CHFEMs with an interface debond defect under the sinusoidal signal.

As shown in Figure 14a, when the measurement distance is 80 mm, the difference between the measured amplitudes of the six PZT sensors in the three CMFEMs and the three CHFEMs corresponding to CMFEM 1 is relatively large. With the increase in the measurement distance, the differences between the PZT sensor responses of the three CMFEMs and the three CHFEMs become smaller. In other words, the influence of the mesoscale heterogeneity of concrete cores and the randomness of coarse aggregate distribution on the measurement made by a PZT sensor becomes nonsignificant and negligible when the PZT sensor is at a certain distance from the PZT actuator. This implies that the mesoscale heterogeneity of concrete cores and the randomness of coarse aggregate distribution only affect the response of PZT sensors in the vicinity of a PZT actuator at a certain level. Similar findings can be found in Figure 14b for the three CMFEMs with interface debond defects and the three CHFEMs corresponding to CMFEM 1 with an interface debond defect.

**Figure 15:**
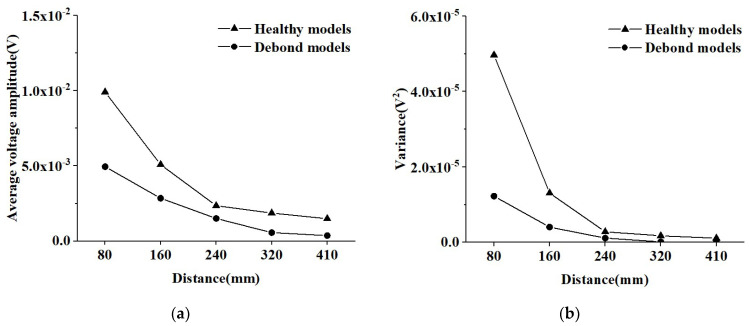
Comparison of average voltage amplitude and variance in the responses of PZT sensors shown in Figure 14 for the three CMFEMs and three CHFEMs with and without debond defects. (**a**) Average voltage amplitude; (**b**) Variance.

Here, the effect of different measurement distances on the response of PZT sensors is further elucidated under sinusoidal signals. The average amplitude and variance in PZT sensors at identical measurement distances in the three CMFEMs and three CHFEMs shown in Figure 14 are analyzed and the results are shown in Figure 15. It can be seen from Figure 15 that the response of PZT sensors close to the PZT actuator is obviously affected when the measurement distance is 80 mm. The variance in the voltage amplitude measured by the PZT sensor in the three CMFEMs and three CHFEMs is the maximum. It is worth noting that when the measurement distance is 160 mm, there is a significant reduction in variance. As the measurement distance increases, the average amplitude and variance gradually decrease. Additionally, the average amplitude and variance in the output signal collected by each PZT sensor in the identical measurement distances of both CMFEMs and the CHFEMs are always much lower than those of the corresponding healthy models. The results indicate again that the interface debond defect plays a dominant role in stress wave measurement.

### 6.2. Responses of PZT Sensors at Different Measurement Distances under the Modulated Sinusoidal Input Signal of CMFEMs and CHFEMs

Here, the influence of the mesoscale hetereogeneity of a concrete core and the randomness of coarse aggregate distribution and of the debond defect on the responses of the PZT sensors of three CMFEMs and the three CHFEMs corresponding to CMFEM 1 under the modulated sinusoidal signal is further distinguished.

**Figure 16:**
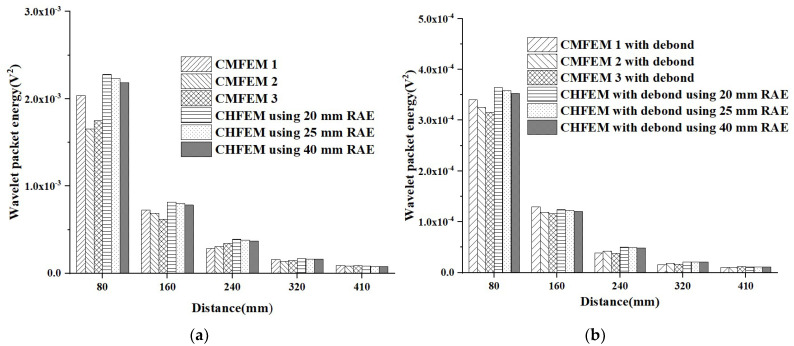
Comparison of wavelet packet energy value of response of PZT sensors between CMFEMs and CHFEMs corresponding to CMFEM 1 with different RAE sizes under modulated sinusoidal signals. (**a**) Healthy CMFEMs and CHFEMs; (**b**) CMFEMs and CHFEMs with interface debond defects.

A wavelet packet analysis of each sensor response is carried out to determine the wavelet packet energy of the output signals collected by each PZT sensor [31], and the results are shown in Figure 16. From Figure 16a,b, it can be seen that the mesoscale heterogeneity of the concrete core and the randomness of coarse aggregate distribution both affect the PZT sensors responses close to the PZT actuator at certain levels. However, the influence becomes unobvious with the increase in the measurement distance. This trend is applicable to both RCFST specimens with and without debond defects. Instead, the influence of the mesoscale heterogeneity of concrete cores and the randomness of coarse aggregate distribution on PZT sensors at the identical measurement distance of specimens with debond defects is much smaller than that of specimens without debond defects no matter the measurement distance is.

**Figure 17:**
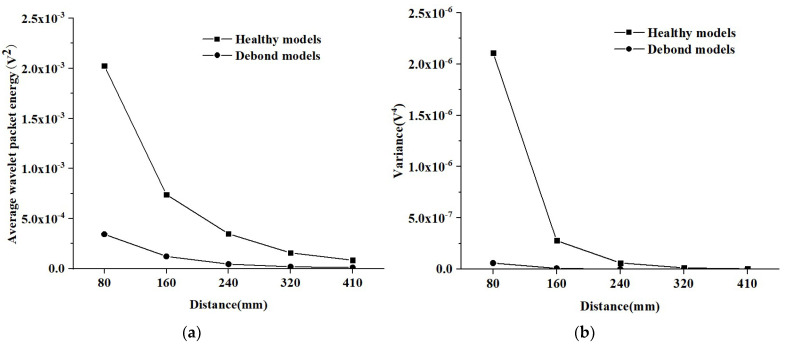
Comparison of average wavelet packet energy and variance in responses of PZT sensors shown in Figure 16 for three CMFEMs and three CHFEMs with and without debond defects. (**a**) Average wavelet packet energy; (**b**) Variance.

Figure 17 shows the average wavelet packet energy value and variance in PZT sensor responses at the identical measurement distances of the three CMFEMs and three CHFEMs shown in Figure 16. Similarly, the variation pattern in the average wavelet packet energy value and variance in output signals collected by the PZT sensor when the measurement distance is consistent with that for output voltage amplitude under sinusoidal shown in Figure 15.

### 6.3. Discussion

As shown in Figure 15 and Figure 17, the variances in both the wavelet packet energy value under a modulated sinusoidal signal and in the amplitude under a sinusoidal signal of PZT sensors in CMFEMs and CHFEMs are relatively large at relatively small measurement distances. When the measurement distance is greater than 160 mm, the variance in the response of the PZT sensors is decreased rapidly. Moreover, even the mesoscale structural randomness and heterogeneity of the concrete core have a certain influence on the response of the PZT sensor close to the PZT actuator; the interface debond defects cause a significant decrease in the signal amplitude measured by the PZT sensors. In summary, the interface debond defect influences the wave fields and the responses of different PZT sensors dominantly. This finding implies that the applicability of the PZT-based stress wave measurement method for interface debond detection in CFSTs even when the concrete core is a typical heterogeneous and random material at the mesoscale.

## 7. Conclusions

Multi-physics and multi-scale CMFEMs composed of a PZT sensor, a PZT actuator and a 2D RCFST cross-section are constructed, and the corresponding CHFEMs with different RAE dimensions are established. The feasibility of using CHFEMs for stress wave field and PZT sensor response simulation and their computational efficiency are illustrated. Then, the difference in the influences of the mesoscale heterogeneity of a concrete core in the form of the randomness of coarse aggregate distribution in a RCFST and of the debond defect on the responses of PZT sensors at different measurement distances are elucidated. Based on the analysis of the numerical simulation results, the following conclusions can be made.

(1) The feasibility and the computational efficiency of the proposed mesoscale homogenization approach for RCFST stress wave fields and PZT sensor response simulation are numerically confirmed. The stress wave fields of the CMFEMs and their corresponding CHFEMs with different RAE dimensions are similar, and the influence of an interface debond defect on the stress wave field is dominant. The concrete core with mesoscale homogeneity in the form of random coarse aggregate distribution in RCFSTs has no obvious influence on the stress wave fields of RCFST specimens.

(2) By comparing the PZT sensor measurement simulation results of healthy CMFEMs and those of the corresponding CHFEMs, the heterogeneity in the form of random concrete core distribution only affects the responses of PZT sensors close to the PZT actuator. The influence of mesoscale heterogeneity on PZT sensor measurement becomes weaker when the measurement distance increases. RAE dimensions have no obvious influence on the PZT responses of CHFEMs. This finding also works for CMFEMs and CHFEMs of RCFST members with interface debond defects.

(3) From the simulation results of the CMFEMs and the CHFEMs, it is clear that the interface debond defects of RCFSTs have a more obvious effect on the response of each PZT sensor when compared with those of a heterogenous and random distribution a concrete core at the mesoscale in RCFSTs. 

(4) The findings from this study imply that the interface debond defect detection method using stress wave measurement from a PZT sensor is reasonable in practice even though the concrete core in RCFST specimens is a heterogenous and random material. The mesoscale heterogeneity and randomness of concrete cores affect PZT sensor responses locally, and the interface debond defect is the dominant factor affecting the PZT sensor measurement no matter what the measurement distance is.

## Figures and Tables

**Table 1:** Material parameters.

Material	Young’s Modulus (GPa)	Poisson’s Ratio	Density (kg/m^3^)
Aggregates	55.5	0.16	2700
Mortar	26	0.22	2100
Steel	207	0.28	7800
PZT	74	0.36	7600

**Table 2:** Comparison of calculation efficiency between MFEM 1 and HFEMs.

	MFEM 1	HFEMs		
		20 mm RAE	25 mm RAE	40 mm RAE
Element number	69,790	29,514	25,561	21,786
DOFs	268,688	113,754	102,866	93,215
Time(s)	5701	2534	2253	1956

## Data Availability

Data are contained within in the article.
